# Valorization of Fruit Waste for Bioactive Compounds and Their Applications in the Food Industry

**DOI:** 10.3390/foods12030556

**Published:** 2023-01-27

**Authors:** Nilesh Prakash Nirmal, Anandu Chandra Khanashyam, Anjaly Shanker Mundanat, Kartik Shah, Karthik Sajith Babu, Priyamvada Thorakkattu, Fahad Al-Asmari, Ravi Pandiselvam

**Affiliations:** 1 Institute of Nutrition, Mahidol University, 999 Phutthamonthon 4 Road, Salaya, Nakhon Pathom 73170, Thailand; 2 ICAR-Central Coastal Agricultural Research Institute, Old Goa 403402, India; 3 Department of Agriculture and Environmental Sciences, National Institute of Food Technology Entrepreneurship and Management (NIFTEM), Sonepat 131028, India; 4 Sargento Foods, 305 Pine Street, Elkhart Lake, WI 53020, USA; 5 Department of Animal Sciences and Industry/Food Science Institute, Kansas State University, Manhattan, KS 66506, USA; 6 Department of Food Science and Nutrition, College of Agriculture and Food Sciences, King Faisal University, P.O. Box 400, Al-Ahsa 31982, Saudi Arabia; 7 Physiology, Biochemistry and Post-Harvest Technology Division, ICAR-Central Plantation Crops Research Institute (CPCRI), Kasaragod 671124, India

**Keywords:** fruit waste, valorization, bioactive compounds, value addition, food fortification, extraction techniques

## Abstract

The fruit production and processing sectors produce tremendous amounts of by-products and waste that cause significant economic losses and an undesirable impact on the environment. The effective utilization of these fruit wastes can help to reduce the carbon footprint and greenhouse gas emissions, thereby achieving sustainable development goals. These by-products contain a variety of bioactive compounds, such as dietary fiber, flavonoids, phenolic compounds, antioxidants, polysaccharides, and several other health-promoting nutrients and phytochemicals. These bioactive compounds can be extracted and used as value-added products in different industrial applications. The bioactive components extracted can be used in developing nutraceutical products, functional foods, or food additives. This review provides a comprehensive review of the recent developments in fruit waste valorization techniques and their application in food industries. The various extraction techniques, including conventional and emerging methods, have been discussed. The antioxidant and antimicrobial activities of the active compounds extracted and isolated from fruit waste have been described. The most important food industrial application of bioactive compounds extracted from fruit waste (FW) has been provided. Finally, challenges, future direction, and concluding remarks on the topic are summarized.

## 1. Introduction

The past decade has unveiled a prodigious advancement in the food industry, making it one of the swiftest-developing segments across the globe. This profuse growth of the sector is conjoined with an array of challenges, among which the two most prominent issues are food safety and food waste management. Baysal and Ülkü [1] reported that, according to estimations, one-third of the food produced annually is either lost or wasted. Consistent with approximations from the Food and Agricultural Organization (FAO), this accounts for a minimum of 1.6 billion tons and is estimated to release a carbon footprint equaling 3.3 billion tons of carbon dioxide [2]. Nevertheless, a major proportion of this waste is attributed to the processing sector. Food waste is termed the by-products or residues derived from the processing of raw material into higher-value products [3]. These wastes are primarily cataloged into two categories corresponding to their origin: animal and vegetable waste. The former classification is known to include wastes that are derived from the meat, seafood, cattle, and dairy processing industries. The latter category includes a large variety of residues depending on the source type [4]. Contrary to other food-processing sectors, the fruit and vegetable industries produce higher volumes of waste, including 25–30% peels, followed by seeds, skins, shells, pods, cores, pulp, pomace, etc. [5]. Its innate perishable attributes, product traits, logistics issues, and disposal are some major apprehensions that make the valorization of this waste a grueling practice. Nevertheless, owing to the incidence of bioactive components, the excesses of the fruit and vegetable industries are considered to be specialized residues. Supervening the mounting consumer demand for higher-value products across all markets, fruit and vegetable residues can be considered to have vast potential that remains unmapped. 

Reviewing the approaches employed in managing wastes, incineration and landfill are the common methods. While incineration involves the production and expulsion of different pollutants, the latter involves the discharge of carbon dioxide and methane, proffering extreme environmental and health impacts [6]. Handling this waste requires a holistic approach encompassing the isolation of the majority of the waste and usage, such as for the synthesis, extraction, or preparation of high-valued compounds [7]. To accomplish this transformation, it is imperative to gain a basic understanding of the countless points of food waste generation, the volume of the waste generated, the nature of waste generated from different sources, and last, but not least, the elementary characterization of waste components. The optimal valorization approach depends on the specific nature and properties of the substance that exist or are attained from individual waste environments. 

This review provides a comprehensive approach to the valorization of fruit waste for bioactive components along with extraction techniques, including Soxhlet extraction, microwave-assisted extraction, enzyme-assisted extraction, high-hydrostatic-pressure extraction, etc., and their applications. This review is structured as follows: the former explains the implications of waste from fruit processing, elaborating on the active components and their antioxidant and antimicrobial activities. The following section involves the elucidation of the methods involved in the extraction of these components and practical utilization of these bioactive components in different areas of food industries. 

## 2. Materials and Methods

This study seeks to offer a comprehensive assessment of the bioactive chemicals that may be extracted from fruit waste and their relevance in the food industry. Electronic searches of the literature, primarily in databases such as PubMed, Google Scholar, Scopus, and ScienceDirect were used to gather published articles for the development of the manuscript. The search terms used were broad and included terms such as valorization, fruit waste utilization, fruit by-products, extraction techniques, bioactive components, etc. A total of 300 scholarly items, including research articles, reviews, books, patents, and other publicly accessible internet sources, were returned by the search procedure. The shortlist included about 200 items that were published after 2000. Due to the scarcity of contemporary studies and their relevance to the chosen topic, articles published before 2015 were also chosen. The selected articles were thoroughly studied and critically analyzed for the preparation of the manuscript. This review outlines a brief introduction, was written based on the PRISMA guidelines, and is correct to the best of our knowledge.

## 3. Fruit Loss and Processing Waste

According to the FAO of the United Nations, about 14% and 17% of the food produced is either lost or wasted globally each year [8]. However, a new report from the World Wide Fund for Nature WWF [9] and Tesco in 2021 revealed that food loss or wastage is around 2.5 billion tons globally each year. This represents an increase of almost 1.2 billion tons from the previously estimated figure of 1.3 billion tons. These updated estimates show that more food is being wasted than was previously thought (33%), with an estimated 40% of all food produced going uneaten. The FAO also stated that food waste would be the third-largest carbon dioxide emitter in the world if it were a nation, after China and the US.

It is projected that the wastage is higher for fruits and vegetables, accounting for approximately 46% (1400 million tons produced are wasted). Fresh fruit and vegetable waste in European Union (EU) households exceeds 17 billion kg annually, or 35.3 kg per person, 14.2 kg of which is avoidable. The average amount of fresh produce purchased by (EU) families wasted is 29% [10]. On the other hand, a survey estimated that Americans throw away $10 worth of fruit every week because it has gone bad or is unusable, which translates to throwing away $520 worth of fruit annually in the United States [11]. According to the Food and Drug Administration (FDA), between 30 and 40% of the food supply is wasted in the United States, which leads to problems [12].

Fruit is lost in fields as a result of crop pests and diseases. Ineffective techniques for fruit harvesting, storage, and transportation also result in fruit loss. Additionally, fruit is also wasted because it is purposefully thrown away in stores, supermarkets, and homes. Thirty-five percent of the wasted food is simply thrown out by supermarkets, shops, and households [13]. Bananas are the most wasted fruit due to brown markings or slight bruising in stores, according to a study from Sweden [14]. It is estimated that 3.7 trillion apples are wasted worldwide each year. Fruit processing generates two different types of waste: liquid waste (juice and wash water) and solid waste (peels/skins, seeds, stones, etc.). Fruit peel waste accounts for between 15 and 60% of the various types of fruit waste that are produced, and it is usually discarded [15]. For several fruits, such as the mango (30–50%), orange (30–50%), pineapple (40–50%), and banana (20%), a significant amount is often wasted. Common fruits, including the mango, banana, orange, watermelon, and lemon, account for between 25 and 57 million tons of waste annually [16].

**Figure 1:**
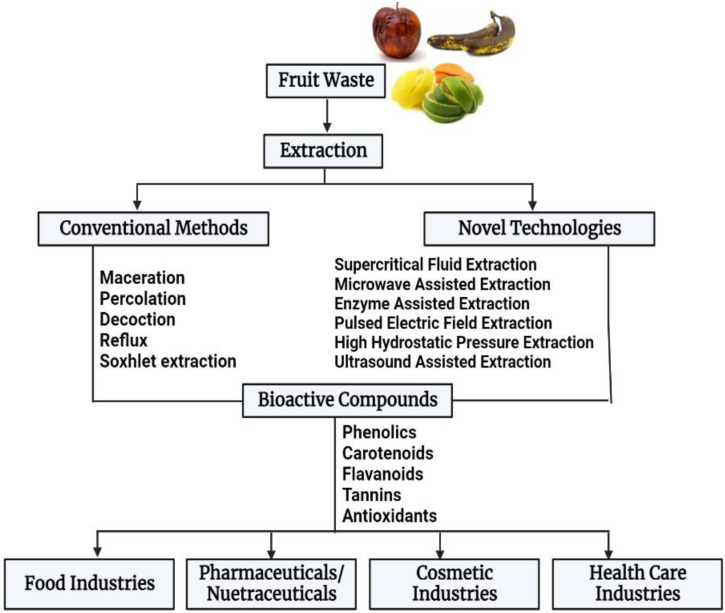
A schematic representation of fruit waste valorization.

These fruit wastes can pose major environmental challenges, such as water and soil pollution, the greenhouse effect, eutrophication, global warming, and other health problems if not effectively handled due to their high biodegradability and fermentability [17]. Although some fruit waste is used as animal feed [18], landfill, incineration, and open burning are the most frequently used methods to dispose of fruit waste [5]. However, these methods or approaches could result in other problems, such as the generation of secondary waste. Additionally, these wastes are renewable and viable resources that could be valorized to create commodities with a high market value following the “circular economy” concept [19]. Therefore, waste recycling and resource recovery are essential for the effective valorization of fruit waste. A schematic diagram of fruit waste utilization is shown in Figure 1.

## 4. Bioactive Compounds from Fruit Waste

Fruit wastes and/or by-products that food agro-industries accumulate are typically made up of underutilized residual biomasses that are rich in various bioactive functional components [20]. Fruit wastes have been researched for the extraction of phenolic compounds, dietary fibers, and other bioactive substances, as they are rich sources of phytochemicals. Peels, pomace, and seed fractions make up the majority of fruit by-products, and they have the potential to be a decent source of bioactive compounds with high added value, such as proteins, dietary fibers, polysaccharides, flavor compounds, and phytochemicals [21]. As a starting point for additional research into the usage of these compounds, researchers and food manufacturers frequently examine the bioactive compounds found in various fruit parts. Therefore, scientific evidence showing the abundance of beneficial components in various fruit parts justifies the consumption of fruit waste in food applications, while also reducing its environmental impact. Studies have shown that sizeable levels of essential nutrients and phytochemicals are available in the peels, seeds, and other parts that are not often utilized, even though most people only eat the pulp of fruits [22]. In contrast to banana peels, which primarily contain gallocatechin, catechin, and epicatechin, the peels of avocado and custard apples have large concentrations of condensed tannins and flavonoids, including procyanidins [22]. However, the predominant compounds found in banana peels are gallocatechin, epicatechin, and catechin [22]. Peels of Prunus cultivars, including the nectarine, apricot, and peach, are abundant in hydroxycinnamates and flavan-3-ols, which may have antioxidant properties [22]. Onion peel is reported to be rich source of flavonoids, including athocyanins, kaempferol, and quercetin derivatives (quercetin diglucoside, quercetin aglycone, and quercetin 4-O-glucoside) [23,24]. Phenolic compounds are secondary metabolites that are among the major classes of significant bioactive compounds with wide-ranging biological effects. In their basic structure, they have one or more aromatic rings, along with one or more hydroxyl groups. Polyphenolic compounds can be divided into several classes, including flavonoids (subclasses: flavonols, flavanones, flavanonols, flavanols, flavones, isoflavones, and anthocyanidins), tannins, phenolic acids, lignans, and stilbenes [25]. According to Wolfe, et al. [26], apple peels can contain up to 3300 mg/100 g of dry matter in terms of their phenolic content. Zadernowski, et al. [27] noted that mangosteen peel and rinds have been shown to contain around two times more total phenolics and phenolic acid than the aril, whereas the mangosteen pericarp has been observed to have a total level of seven primary xanthones that is eight times greater than that in the aril [28]. It was formerly reported that the total phenolic content of mango peels is roughly 13–47% higher than that of the flesh and 32% higher than that of the seeds [29,30]. The phenolic content of papaya peels is about 1.2 times higher than that in the seeds [31]. On the other hand, the biochemical indices of the crude fiber of papaya seeds are much greater than those of the pulp and peel, although they have a lower total fiber content [32,33]. Passion fruit seeds and pulp are known to have much higher total phenolic and flavonoid concentrations, although they have lower total dietary fiber [34]. Both the peel and pulp of the dragon fruit contain a considerable amount of pectic compounds; however, the peel exhibits a higher level of pectic compounds than the pulp [35]. According to reports, tomato seeds contain a variety of bioactive substances, including bioactive peptides, flavonoids, carotenoids, pectin, and vitamins (tocopherol) [36]. Guava seeds are also reservoirs of bioactive components, such as fatty acids, including palmitic, linoleic, and oleic acid, as well as vitamin C, vanillin, and vanillic acid [37]. An unpalatable byproduct of the fruit is the Jamun seed. However, its high concentration of phytochemicals makes it a valuable source of nutraceuticals. The presence of phytochemical components, such as phenols, tannins, flavonoids, saponins, triterpenoids, steroids, and alkaloids, in the Jamun seed is associated with its bioactivity [38].

It has been previously reported that pineapple skin contains substantially more lutein, α-carotene, and β-carotene than the core [39]. Notably, it has been reported that both the fresh and dried pulp of rambutan has higher levels of ascorbic acid than the fruit’s peel and lower levels of carotene [40]. Contrarily, despite the content of total carotenoids derived from mango peels being much higher than that found in the kernel, they are poorer in terms of their total phenolic content and antioxidant activity [41]. Among plants, raw grape leaves (16.19 mg/100 g) are considered a key source of β-carotene [42], and β-carotene is widely utilized in the food additive, cosmetics, health care, and pharmaceutical industries. Markedly, it has demonstrated several advantages, including improved human immunity, antioxidant activity, protection against various malignancies, and a reduced risk of cardiovascular illnesses due to its ability to manage cholesterol levels [43]. Along with β-carotene and lutein, lycopene is one of the primary carotenoids extracted from tomato waste [44]. Lycopene is a phytonutrient with a significant impact on human health and it has long been recognized for its range of biological properties, including antioxidant, anti-inflammatory, etc. [45,46]. Lutein is a yellow–orange carotenoid that is a member of the xanthophyll family and is frequently found in fruits [47]. The main anthocyanin found in many fruits is cyanidin 3-O-glucoside, which is the most prevalent anthocyanin in plants and has been linked to anti-obesity, anti-inflammatory, antioxidant, and anti-tumor characteristics [48,49]. Non-anthocyanin phenolic chemicals, such as flavonols (myricetin, quercetin, and kaempferol) and flavones (luteolin and apigenin), are a promising family of natural food colorings. In fruits, they are present mainly as quercetin [50]. For instance, elderberry contains a significant amount of quercetin derivatives, and quercetin is reported to have positive benefits on health; typically, they are well-known for their antioxidant, anti-obesity, and anti-inflammatory properties, and can be used in preventing cardiovascular illnesses [51,52]. Apple pomace is typically discarded as waste material in processing industries after the juice has been extracted. This waste can, however, be an excellent source of nutritional fiber. According to reports, apple peel contains more dietary fiber than apple pulp. The amounts of soluble and insoluble dietary fiber in apple pomace are 15% and 36%, respectively [53]. Apple seeds are also reported to be a rich source of bioactive compounds [54]. The pomace powder of blackcurrants, red currants, gooseberries, rowanberries, and chokeberries is also reported to have a high fiber content (>550 g/kg) [55]. The total amount of dietary fiber found in grape pomace is around 78%, of which 9.5% is soluble and the remaining 68% is insoluble [56]. Ajila and Prasada Rao [57] evaluated the total dietary fiber in mango peels and revealed their content to be 40–72%, with glucose, galactose, and arabinose being the main neutral sugars in the soluble and insoluble dietary fibers. The dietary fiber concentrations in the pulp and peels recovered as a byproduct of the extraction of peach juice range from 31–36% (dry weight), with 20–24% insoluble dietary fiber making up the majority.

## 5. Extraction Techniques

Presented with a wide range of bioactive chemicals and a multitude of plant species, it is essential to develop a standardized and comprehensive screening method for extracting compounds that are advantageous to human health. The use of bioactive chemicals in several industrial fields, including the food, chemical, and pharmaceutical industries, indicates the need for the most efficient and standardized technique to extricate bioactive compounds from plant materials. The extraction of bioactive components from plant matrix can be accomplished using a variety of extraction techniques, and the choice of an appropriate technique alters the cost, duration, and accessibility of the procedure. An efficient extraction approach should be able to target bioactive compounds from the plant matrix, have high selectivity towards analytical procedures and bioassays, and provide a robust and reproducible method that is free of fluctuations in the sample matrix [58]. The bioactive components can be extracted by using conventional or novel extraction techniques. Some of the widely used extraction techniques in the food industry, along with their advantages and disadvantages, are discussed in Table 1.

### 5.1. Conventional Methods

The polarity/ionic strength of various solvents in use, along with the usage of heat and/or mixing, are the key factors influencing the effectiveness of conventional extraction processes [87]. Soxhlet extraction, maceration, solvent extraction, reflux extraction, etc. are examples of traditional extraction techniques. In maceration, the sample is ground into fine particles to enhance its surface area and facilitate solvent mixing (water or an organic solvent). The solvent is then combined with the ground materials, followed by continuous agitation, and contaminants are later removed using filtration. The relatively simple extraction technique of maceration has the drawbacks of a lengthy extraction period and poor extraction effectiveness. However, thermolabile components could be best extracted using maceration. Another extraction method that is more effective than maceration is percolation. It is an unceasing process that utilizes a special piece of machinery called a percolator, in which the saturated solvent is continuously changed out for a new solvent. The percolator is typically filled with dried powdered samples, which are then mixed with boiling water and macerated for a few hours. To obtain concentrated extracts, evaporation is carried out after the completion of extraction [88]. Another common extraction method is called decoction, which involves boiling the crude aqueous extract to a pre-determined volume for a specific amount of time to extract the heat-stable components. The liquid is allowed to cool and is strained or filtered after it settles. The method can be used to extract water-soluble components. It should be noted that this process is ineffective for materials that are sensitive to heat and light, and volatile or thermolabile substances cannot be obtained using decoction. In addition, mass transfer and kinetic effects must be taken into account [64]. Compared with percolation or maceration, reflux or solid–liquid extraction is more effective, uses less solvent, and has a shorter extraction time [87]. This procedure involves mixing a dry sample with the solvent in a heated, agitated jar. Better mass transfer and contact efficiency between the solvent and the treated matrix are gained when the vapors are allowed to condense and trickle back into the flask. Compounds with high thermolability cannot be extracted using this method [75]. Soxhlet extraction has long been the most extensively operated method for concentrating analytes and separating bioactive components from natural materials. Utilizing the principles of reflux and siphoning to constantly extract the bioactive component with fresh solvent, the Soxhlet extraction process combines the benefits of both percolation and reflux extraction. Compared with maceration or percolation, the Soxhlet extraction process has a high extraction efficiency, takes less time, and has lower solvent consumption. However, the high temperature and prolonged heat exposure could increase the thermal degradation of the bioactive compounds [69]. 

### 5.2. Novel Emerging Methods

Numerous studies have demonstrated the effectiveness of traditional extraction techniques, including the Soxhlet extraction and maceration processes. However, using such techniques requires the use of a lot of time, energy, and solvent. There are alternative extraction methods that have faster extraction times, higher selectivity, and higher efficiency, and use less solvent to overcome the disadvantages of conventional extraction procedures. These procedures are referred to as non-conventional or green extraction methods, or novel extraction methods [89]. The application of novel technologies, such as ultrasound and pulsed electric fields, to grapes has increased the polyphenol content by 32–23% and decreased the energy consumption by 17.6 fold [90]. Some of the promising non-conventional extraction techniques are discussed below.

#### 5.2.1. Supercritical Fluid Extraction (SFE)

Since Hannay and Hogarth’s discovery of supercritical fluid in 1879, it has been utilized for extraction purposes, and in 1964, it was employed in the food industry to decaffeinate coffee [58]. SFE has gained popularity in recent years as a method for extracting bioactive components from plants at atmospheric temperatures while avoiding thermal denaturation. Supercritical fluid (SF) is used as the extraction solvent in supercritical fluid extraction. A substance can only reach the characteristic supercritical state if it is subjected to pressure and temperatures beyond its critical point. Supercritical fluid exhibits liquid-like density and solvation power, and gas-like viscosity, surface tension, and diffusion characteristics in its supercritical state. These characteristics allow for faster and higher-yielding chemical extraction. Due to its low critical temperature (31 °C), inertness, low cost, and non-toxicity, supercritical carbon dioxide is frequently utilized in SFE. The main aspects that affect the extraction efficiency of SCF extraction are the process temperature, pressure, flow rate, and sample volume [73]. The efficiency of SFE in extracting bioactive components from plant matrices has been reported in various studies [91]. SFE can be used to extract alkaloids, such as Pyrrolidine [92], caffeine [93], Olchicine [94], and Vinblastine [95], essential oils [96], terpenes [97], flavonoids [98], and phenolic compounds [99].

#### 5.2.2. Microwave-Assisted Extraction (MAE)

The microwave-assisted extraction technique is regarded as a novel practice that uses microwave radiation to extract soluble compounds into a fluid from a variety of matrices. Electromagnetic radiation with frequencies between 300 MHz and 300 GHz is known as microwaves [100]. They are composed of electric and magnetic fields that oscillate perpendicular to each other. The microwave heating principle relies on the dipole rotation and ionic conduction mechanisms. The resistance of the medium to the flow of ions during ionic conduction causes heat to be produced, whereas the electromagnetic field change brought on by microwave radiation will frequently cause changes in molecular orientation, thereby producing heat by molecular friction [101]. The high extraction yield in MAE is due to the synergistic effect of the heat and mass gradients. MAE involves three stages; first, the solvent’s penetration into the plant matrix, followed by the breakdown of the components by electromagnetic waves, and the transport of the solubilized components from the insoluble matrix to the bulk solution. Finally, liquid and residual solid phase separations are performed [102]. MAE can be used to extract a variety of bioactive components, such as flavonoids [103], isoflavone [104], saponins [105], piperine [106], carotenoids [107], terpenes [108], essential oils [109,110], polysaccharides [111], etc.

#### 5.2.3. Enzyme-Assisted Extraction (EAE)

The phytochemicals in plant matrices can be either disseminated in the cell cytoplasm or found attached to the polysaccharide–lignin network by hydrogen or hydrophobic interactions, making the compounds inaccessible for extraction using a solvent in a typical extraction technique [102]. It has been suggested that enzymatic pre-treatment is a novel and efficient method for releasing bound molecules and improving the total yield. To acquire bioactive chemicals, enzyme-assisted extraction (EAE) can be used as a pre-extraction or extraction procedure. The plant cell wall is destroyed, releasing the bound bioactive chemicals attached to the lipid and carbohydrate chains [76,77]. The major enzymes that are used in EAE are cellulases [112], pectinase [113], hemicellulase [114], amylase [115], glucosidase [116], etc. EAE can be used for extracting bioactive components such as anthocyanins [117], polyphenols [118], oleoresin [119], flavonols [120], terpenes [121], carotene [122], etc.

#### 5.2.4. Pulsed Electric Field Extraction (PEFE)

Pulsed Electric Field Extraction (PEFE) promotes mass transfer during extraction by breaking down membrane structures, thereby considerably enhancing the extraction yield and decreasing the extraction time. The cell membrane experiences an electric potential when it is deferred in an electric field, and when the electric potential exceeds a critical value, repulsion between charge-carrying molecules creates pores in vulnerable regions of the membrane, dramatically increasing permeability. The field strength, pulse count, specific energy, and treatment temperature are all factors that affect PEFE treatment [123]. Due to its energy efficiency, PEFE processing is a feasible technique for the food, pharmaceutical, and biotech industries. PEFE can be used for extracting polyphenols [80], flavonoids [79], proteins [81], anthocyanins [82], and carbohydrates [78]. 

#### 5.2.5. High-Pressure Extraction

High-pressure extraction, also known as pressurized liquid extraction (PLE), accelerated solvent extraction, enhanced solvent extraction, or pressurized fluid, involves using a high pressure to keep solvents in the liquid state above their usual boiling point. The high pressure maintains solvents in a liquid condition above their boiling point, leading to high lipid solubility, high lipid solute diffusion rates, and high solvent penetration of the matrix [124]. Compared with other procedures, PLE significantly reduces the extraction time and amount of solvent used and has excellent repeatability. High-pressure extraction has been effectively used by researchers to extract an array of bioactive components, such as phenolic compounds, carotenoids, flavonoids, pectin, etc. [83].

#### 5.2.6. Ultrasound-Assisted Extraction (UAE)

With frequencies between 20 kHz and 100 MHz, ultrasound is a specific kind of sound wave that is not audible to humans. Similar to other waves, it compresses and expands the medium as it travels through it. This process causes a phenomenon known as cavitation, which denotes the formation, expansion, and collapse of bubbles. This event releases a significant amount of energy, which causes cell rupture [86]. The process of applying intense ultrasonic waves for extraction is known as ultrasound-assisted extraction (UAE). The technology is renowned for its ease of use and relative affordability when compared with other traditional extraction methods. Moreover, UAE has lower solvent usage, a shorter extraction time, and lower energy consumption. Sonication can also facilitate efficient mixing and quicker energy transfer. UAE is an efficient technique for the extraction of bioactive compounds, such as polyphenols, flavonoids, anthocyanins, etc. from various plant matrices [58,84,85,86]. 

## 6. Bioactivities of Active Compounds Extracted from Fruit Waste

The further utilization of parts of fruit by-products is only possible with exploratory studies on the bioactivities of their constituent compounds. Scientific studies on the amounts and the functions of these active constituents serve as an insightful reference and a justification to researchers and manufacturers for the successful extraction of these components. Fruit by-products, as their source, are stated to be plentiful in high-value compounds, such as bioactive compounds that are considered to have an effect on human health owing to their biological properties, including anti-inflammatory, antioxidant, antimicrobial, antimutagenic, etc. [7,125]. The physiological activity exhibited by the array of by-products of fruits is due to the synergistic action of these distinct compounds [126]. 

### 6.1. Antioxidant Activity

Antioxidants are defined to be compounds that can inhibit or adjourn oxidation and thereby diminish the concentrations of transition metal ions or free radicals [127]. The consumption of and introduction of food products with these compounds to the diet can help in maintaining the antioxidant status and also control the development of chronic diseases, such as cancer, cardiovascular issues, etc. The antioxidant potential of fruit coproducts is dependent on several factors, with the main points under consideration including the fruit type and by-product involved (e.g., peel, seeds, stems, pulp, etc.). Taking the latter point under consideration, various studies have compared the potential of each type of by-product obtained from fruits (Table 2). The pre-eminence of peels in the antioxidant potential of different types of by-products compared with seeds and other parts has been reported in different studies [128,129]. Apples are known for their abundant reserves of phenolic substances, among which most of the active components are concentrated in the apple pomace [130]. The antioxidant properties of apple by-products are attributed to the different classes of these phenolic components and their oxidation products. The dominance of apple by-products in terms of antioxidant potential over certain other fruits was reported by Duda-Chodak and Tarko [128]. Maximum antioxidant activity was found in the peels of apples (7925 mg Trolox × 100 g^−1^ d.w.), followed by white grapes (6944), and the seeds of orange and Idared apples. The same trend has been observed among the seed portions with the highest antioxidant potential attributed to different varieties of apples. Peschel, et al. [131] also reported the high antioxidant potential of apple residues in comparison with pear and strawberry residues obtained after juice production. The citrus genus is one of the most cultivated and utilized groups of fruits, which accounts for 50–60% of citrus by-products, including peels, seeds, pulps, stones, etc. The by-products of the processing industries are reported to have high volumes of polyphenols, mainly flavonoids and phenolic acids, compared with the edible portion [132]. The antioxidant potential of citrus parts is attributed to these active components, mainly flavonoids [133]. The potential of various species is decidedly reliant on the cultivar, species under consideration, type of by-product, and harvesting conditions [129]. Chen, et al. [134] reported variations in the phenolic and flavonoid contents of dried citrus peel derived from Citrus reticulata from different geographical areas. According to the difference in the zone, there was a variation of 42–51.8 mg GA/g in the total phenol content and 14–31.9 mg/g in the flavonoid content of the species. Deviation in the polyphenol content of citrus fruits based on the type of by-product was reported by Xi, et al. [135], showing the superiority of lemon peels over the seeds. Similar to this, many reports have reported a higher phenolic content in the peels of fruits (papaya, passion fruit, mango, and mangosteen) when compared with the pulp, stones, or seeds [136,137,138]. The total phenolic content of papaya peels was 1.2 times higher than that of seeds, which was attributed to the superior antioxidant potential [31]. Added to these, some reports illustrate the fact that the peel fractions of some fruits possess more active constituents and higher antioxidant activity than the pulp portions. Li, et al. [139] elaborated on the possibility of higher antioxidant activity of pomegranate peels than that of the edible portions. Palanisamy, et al. [140] considered the antioxidant potential of rambutan and described rambutan peels as a potent source of natural antioxidants owing primarily to the presence of phenolic acids and ellagitannins. 

### 6.2. Antimicrobial Activity

Agents that influence the elimination or inhibition of the growth of pathogenic or spoilage microorganisms are termed antimicrobial agents. As microbial growth and activity are prevailing conditions that affect food products’ quality as well as safety, the significance of these components or agents is illustrious. Antimicrobial components are known to be present in different parts of plants, such as peels, fruits, pods, leaves, seeds, etc. (Table 3). These constituents penetrate the cell membrane, causing lysis and protection against pathogenic microbes. Good inhibitory activities of plum, elderberry, and Italian red grape by-products against potential pathogenic strains were reported by Coman, Oancea, Verdenelli, Cecchini, Bahrim, Orpianesi, Cresci and Silvi [147]. Elderberry skin and seed extracts were reported to have a good inhibitory effect against the growth of *Bacillus cereus*, with an inhibition zone of almost 20 mm. A moderate inhibitory effect of all fruit extracts was observed against *L. monocytogenes* and they had very little inhibitory activity against the probiotic strains. Similarly, the by-product extracts of mandarins also showed significant inhibitory effects on both Gram-positive and -negative bacteria, with inhibition zones of 16.1 and 17 mm [150]. Gunwantrao, et al. [151] also reported the effectiveness of orange and pineapple peel extracts against the pathogenic bacterial strains *Klebsiella pneumonia*, *Pseudomonas aeruginosa*, and *Bacillus subtillis*, with a maximum zone of inhibition. Muscadine grape polyphenols exhibited strong antibacterial activities against a broad range of food-borne pathogens, mainly *Staphylococcus aureus*. A reported decrease of nearly 5 log10 CFU/mL in cell viability for *S. aureus* was observed in a 6 h period with lysis [152]. In the same way, various researchers have studied the antibacterial potential of several other fruit by-products, including those of bananas [153], mangoes [154], cloudberries, and raspberries [155], against *S. aureus* growth and activity. The potent antimicrobial activity of mango kernel extracts was ascribed to the incidence of phytochemicals, including flavonoids, terpenes, coumarins, and tannins [154]. There is a correlation between the by-product extract concentration and the inhibition efficiency [156]. Likewise, anti-fungal and yeast growth-inhibiting properties of bioactive components in banana peel were reported by Aboul-Enein, et al. [157]. The antimicrobial potential of these peels was ascribed to the presence of tannins and phenolics in the sample. Analogous to antioxidant activity, the antimicrobial potential of fruit by-products is reliant on certain elements, among which the type of residue involved is an imperative aspect. Owing to variations in the chemical compositions of different parts of fruits, there are differences in their bioactivity potentials. Kanatt, et al. [158] reported a disparity between the antimicrobial activities of pomegranate peel and seeds. Pomegranate peel extract exhibited exceptional antioxidant activity against *Staphylococcus aureus* and *Bacillus cereus*, while the seed extract did not have any substantial activity. This was attributed to the variance in the form and quantity of bioactive compounds present in both tissues. Even though there have been many studies concentrating on the possibilities of by-products as antimicrobial agents, the principal components responsible for such activity have not been evaluated in many cases. 

### 6.3. Other Properties

Apart from the major targets of the bioactive components present in different by-products of fruits and vegetables, they have other key physiological properties including anti-inflammatory, anti-carcinogenic, anti-melanosis, cardioprotective effects, etc., attributed to these active constituents. Accordingly, it is necessary to elucidate these properties in detail. Pomegranate by-products exhibit anti-cancer, anti-inflammatory, and anti-aging activities with the incidence of punicalagin and ellagic acid as bioactive constituents [162]. Similar to these, citrus by-products are also known for their anti-cancerous and anti-inflammatory properties owing to different bioactive constituents, including flavanones, flavones, and anthocyanins [129]. In addition to these, the major constituents of citrus oil, the terpenes, citral aldehydes, and esters, have a major role in operative therapy for cancer-related issues [163]. Melanosis is the harmless, but unappealing, external discoloration of shrimp, crab, or lobster and is instigated by the enzymatic oxidation of colorless phenols into quinones. The active components of the by-products of olive reportedly help in the diminution of melanosis in shrimps [164]. Phenolic compounds present in these olive by-products are responsible for blocking the progression of discoloration in shrimps. 

## 7. Application of Bioactive Compounds in the Food Industry

Agricultural production currently creates substantial amounts of organic waste from agricultural wastes and the industrialization of the output, such as food industry waste. This industrialization process engenders large quantities of co-products that are difficult to preserve because of their chemical and physical–chemical properties. Historically, these co-products have been utilized for animal food or compost. In their composition, however, it is likely that several compounds with high added value will be identified that, after undergoing an appropriate conversion process, could be transformed into marketable products as ingredients for the development of new food products to obtain the benefits of the vast quantity of potentially valuable compounds that they contain. Some of the food industry’s by-products include fruits, skins, seeds, and membrane residues that have been discarded. These fractions are rich sources of many bioactive compounds, such as dietary fiber (pectin, cellulose, hemicellulose, and lignin), minerals (potassium, calcium, magnesium, and selenium), organic acids (citric, oxalic, and malic acids), vitamins (vitamin C, thiamine, riboflavin, and niacin), phenolic acids (chlorogenic, ferulic, and sinapic acids), flavonoids (hesperidin, narirutin, didymin, hesperetin, and diosmin), terpenes (limonene), carotenoids (lutein, β-carotene, and zeaxanthin), etc. [15,165,166,167,168]. Numerous health benefits have been linked to these bioactive substances, including antioxidant, antibacterial, anti-inflammatory, anti-hypertensive, neuroprotective, and antiallergenic activities [166,169,170,171]. As a result, the creation of several products employing by-products from agro-industrial waste is gaining interest in the food industry. This section discusses the use of some of these compounds for food fortification and food preservation. 

### 7.1. Food Fortification

The consumption and processing of a variety of fruits, such as apples, mangos, grapes, and citruses, generate numerous by-products that frequently contain a high concentration of useful bioactive compounds. One of the biggest by-products of processing fruits is the fruit pomace. Fruit pomace can be used in food items as a cost-effective, low-calorie bulking agent to replace some of the sugar, fat, or flour. It frequently improves food functionality by enhancing emulsion stability and water and oil retention [172]. Fruit pomace often combines the usual fruity and baked taste and aroma of the finished products to improve the aroma and flavor of baked goods. By using 30% (*w*/*w*) apple pomace, researchers developed several high-fiber, functional baked and extruded snacks. The product’s chemical composition remained unchanged when compared with the control [173]. In another study, up to 20% (*w*/*w*) mango peel powder enhanced the soluble dietary fiber and hardness while reducing spreading in soft dough biscuits. Contrarily, it was discovered that adding mango peel powder up to 30% (*w*/*w*) improved the nutritional value of cookies without impairing their sensory or textural qualities [174]. Similar to bakery products, the use of fruit pomace in meat products has also been investigated by several researchers. To increase the dietary fiber content of meat products, fruit pomace has been added to different meat products. For example, apple pomace in meat could make up for the lack of fiber in our diets. A study developed beef patties with 2–8% apple pomace as a beef substitute [175]. The water-holding capacity, cooking yield, meat emulsion stability, and textural qualities, such as the firmness, toughness, and hardness, of patties were improved with higher apple pomace powder incorporation. However, only the addition of apple pomace powder up to 6% was deemed acceptable based on a sensory examination of the patties. Similarly, it was reported that red grape pomace could enhance the color stability and acceptability of pork burgers by reducing lipid oxidation. When the percentage of fruit pomace replacement exceeded 6%, a decrease in hardness and cohesiveness was found [176]. 

Fruit pomaces are sometimes also used in dairy products as a natural texturizer and stabilizer. Apple pomace was added to skimmed milk in three different concentrations (0.1%, 0.5%, and 1%) and then fermented at 42 °C by *Lactobacillus bulgaricus* and *Streptococcus thermophiles*. The outcomes showed that adding 1% pomace caused a higher onset pH and quicker gelation. Additionally, after 28 days of storage, yogurt supplemented with fruit pomace showed enhanced cohesion and consistency [177]. Similarly, the addition of 3% pomace to stirred yogurt caused a noticeably lower level of syneresis and an increase in the matrix’s stiffness, cohesion, and viscosity [178].

Citrus fruits (orange, lemon, mandarin, and grapefruit) are also among the most widely grown crops that produce a huge quantity of co-products, such as peel and pulp (seeds and membrane residues). Soluble dietary fiber and insoluble dietary fiber, which can be found in citrus co-products, are outstanding sources of dietary fiber. Several studies reported very intriguing technological–functional properties of citrus co-products due to their high dietary fiber content, including their water-holding capacity (WHC), oil-holding capacity (OHC), swelling capacity (SC), foam capacity (FC), and emulsion capacity (EC). Citrus co-products can be used to increase the dietary fiber content or serve as a fat substitute in meat products. In this regard, a study examined the impact of adding lemon fiber at 2, 4, and 6% on the amount of cholesterol in low-fat beef burgers. The researchers discovered that adding lemon fiber lowered the amounts of cholesterol and saturated fatty acids in a concentration-dependent manner [179]. Similar to this, low-fat Frankfurt sausages were supplemented with various amounts of citrus fiber (1, 2, and 3%). According to these authors, the sausage samples that had citrus fiber added to them had reduced levels of saturated fatty acids and better water-binding properties [180]. Citric acid, one of the by-products of kiwi processing, can prevent browning and maintain color characteristics during the osmotic dehydration of kiwifruit slices [181]. Another compound of interest from kiwi is Actinidin. Actinidin has potential applications as a cost-effective coagulant in milk. According to a study, kiwi extract caused a casein clot to form that was isolated from the serum and remained stable for up to two months at room temperature [182].

Beyond fulfilling fundamental nutritional needs, bioactive substances have positive health effects on the host. Due to the GRAS (Generally Recognized as Safe) status of medicinal herbs, extracts, or essential oils, they can be added to a variety of food products. The effect of flaxseed extract, which is high in linolenic acid, lignans, and fiber, on the development and survival of kefir-isolated lactic acid bacteria was demonstrated in an in vitro investigation by [183]. The growth of *Lactobacillus kefiranofaciens* DN1, *Lactobacillus bulgaricus* KCTC3635, *Lactobacillus brevis* KCTC3102, and *Lactobacillus plantarum* KCTC3105 was reported to be considerably higher after treatment with crude flaxseed extract than that in the control. Similarly, the characteristics of kefir drinks that had been supplemented with yam, sesame seed, and bean extracts were examined [184]. Upon the application of different concentrations (25, 50, and 75%) of these extracts, the results demonstrated that the fermentation of yam, sesame, and bean extracts by water kefir grains was acceptable for the preparation of fermented vegetable beverages. In addition, the formulation enhanced with 50% beans was the finest base for producing kefir beverages, as well as a protein-rich beverage. To partially replace the fat in an emulsified meat system, the impact of orange peel addition, employed as a fat substitute, on the oxidative stability of low-fat beef burgers was examined [185]. The authors claimed that the samples in which orange peels were used as a fat replacer had peroxide levels that were lower than those of a control sample, with reductions of >90%. Given that its dietary fiber can aid in regulating colon bacterial populations and lower the synthesis of mutagens following the fermentation of food chemicals by intestinal bacteria, the prebiotic capacity of kiwis is one of their most researched characteristics [186]. It has been demonstrated that eating cooked starch with kiwis delays the digestion and absorption of carbohydrates and has hypoglycemic effects [187]. The use of kiwi seed oil as a component of dietary supplements intended to lower cholesterol and prevent obesity has been suggested. It would have an anti-inflammatory effect, enhance the intestinal flora, lower blood sugar levels, and promote a lipid-lowering effect [188,189].

### 7.2. Food Preservation

Bioactive compounds such as phenolics, which comprise terpenes, aliphatic alcohols, aldehydes, ketones, acids, anthocyanins, and isoflavonoids, are the most important group of chemicals with antimicrobial activity [190,191]. The fundamental function of phenolics is in plant defense against biotic and abiotic stressors, pathogens, and pests [192,193,194]. Flavonoids are a wide category of phenolic compounds found in several fruits, vegetables, and roots, among other foods [195,196]. The subclasses of flavonoids include flavanones, flavonols, flavones, flavonols, isoflavones, and anthocyanidins [197]. 

Grape seed extracts are by-products of winemaking or grape juice production and are high in proanthocyanidins and other phenolic compounds [198,199,200]. The use of the Isabel and Niagara varieties of grape seed extracts as natural antioxidants in amounts of 40 and 60 mg, respectively, delayed the lipid oxidation of processed, cooked, and refrigerated chicken meat for 14 days, with effects comparable to those of the synthetic antioxidant butylated hydroxytoluene (BHT). Similarly, the combination of grape extracts with vacuum packaging has been shown to be an effective method for enhancing the lipid stability of cooked chicken [201]. Several studies have also reported the antibacterial effectiveness of grape extracts against lactic acid bacteria, foodborne pathogens, and wine-rotting yeasts [202,203,204,205,206,207]. Grape seed extracts suppressed the growth of foodborne pathogens, such as *Staphylococcus aureus*, *Salmonella* sp., *Escherichia coli*, *Listeria monocytogenes*, and *Campylobacter* sp. [208,209,210]. Depending on their composition, citrus peels are abundant in several nutrients that serve as functional and antimicrobial compounds. These by-products contain secondary metabolites, such as terpenoids, carotenoids, coumarins, furanocoumarins, and flavonoids, particularly flavanones and polyethoxylated flavones [211]. The addition of citrus oil in combination with milder heat treatments has been reported to have an impact on the control of spoilage bacteria in apple and orange juices [212]. On the other hand, mango seed biowaste has also been characterized by a high concentration of bioactive components, including phenolic compounds, carotenoids, and vitamin C [213,214]. A study reported an array of antibacterial properties for mango seed ethanolic extracts and reported their efficacy against Gram-negative bacteria [215]. Various mango peel extracts were evaluated for their antibacterial effects against Gram-positive *Staphylococcus aureus* and Gram-negative *Pseudomonas fluorescens*. Different levels of antibacterial activity were present in the extracts against both. In general, Gram-positive bacteria are more sensitive to natural substances than Gram-negative ones. The peel of the Langra mango variety showed the greatest zone of inhibition for both organisms when it was extracted with 70% ethanol and 80% acetone. Due to the existence of various cell wall architectures, Gram-positive and Gram-negative bacteria exhibit diverse antimicrobial properties. More potent antibacterial substances may include those that can fluidize the membrane and successfully diffuse the lipid bilayer [216]. Avocado peels and seeds contain many bioactive components, including phenolic acids, condensed tannins, flavonoids (including procyanidins and flavonols), and hydroxybenzoic and hydroxycinnamic acids [217,218,219]. Studies have demonstrated the antibacterial action of avocado seed extract components against microorganisms. A recent study demonstrated the biocidal impact of avocado seed extracts against *L. monocytogenes*, suggesting that this action was caused by an increase in cell membrane permeability. Avocado seed ethanolic extract (104.2–416.7 μg/mL) was found to exert antibacterial effects against *L. monocytogenes* (*Staphylococcus epidermidis*, and *Zygosaccharomyces bailii* [220]. Table 4 depicts some additional food-preservation effects from food waste. 

## 8. Challenges and Future Direction

Fruit and vegetable wastes from the agri-food sector are produced in enormous quantities and, due to their high moisture content and microbial load, can lead to significant environmental damage. Bioactive components could degrade quickly, even with the slightest alterations in extraction techniques. For instance, a 22% reduction in phenolic content was reported in strawberries due to the influence of extraction parameters such as temperature and pressure [227]. Extraction process parameters, such as pressure, temperature, light, pH, etc., can cause rapid variations in the quality and quantity of the extracted bioactive compounds, thereby facilitating losses [75]. Therefore, it is necessary to ensure conditions that will stabilize the bioactive components before and after extraction. Choosing an appropriate optimized extraction technique is critical as it decides the final quality of the bioactive compound. Today, the utilization of natural bioactive compounds in the food business has been hampered by the lab-intensive and lengthy extraction and isolation methods. As technology advances, new rapid and efficient technologies for extracting and separating natural compounds emerge that can yield high-quality extracts with a better yield and reduced time and usage of solvents. However, the higher cost associated with these novel technologies and the difficulties in scaling up to industrial standards remains a major challenge. There are numerous potentials for employment and revenue growth in the market for bio-based products. In recent years, these might account for up to 10% of chemical industry penetration and the creation of nearly 200,000 jobs in the United States alone. By 2020, the global nutraceuticals market was projected to increase by 8% annually and reach a value of USD 263 billion [148]. However, these encouraging prospects must overcome significant obstacles, such as the perishable nature of fruit and vegetable waste, logistical problems brought on by the dispersed generation of fruit and vegetable wastes, the composition of fruit and vegetable wastes, which often exist as complex mixtures that increases the cost of extraction, etc. 

In terms of industrial application, these chemicals are known to be used mostly in the cosmetic and food industries [228]. The use and application of the extracted bioactive compounds in the food industry depend on the amount of bioactive component (BC) extract, the mode of addition into the food matrix (crude or powder), and external factors, such as heat, light, pressure, etc., during food processing. Additionally, before advertising and employing the bioactive component for consumer use, the extracts must undergo an in vivo analysis to validate their bioactivity, stability, safety, and bioavailability. Moreover, further research is needed to determine the best extraction procedures to meet the requirements for food fortification and other applications.

## 9. Conclusions

The study of fruit waste valorization has gained recent significance as it can be used as an important tool to meet sustainable development goals and help to combat the carbon footprint and greenhouse gas emissions that are mostly caused by these wastes. Although some fruit by-products contain even more bioactive ingredients than the original fruit itself, they are typically seen as waste and thrown away. The high quantity of co-products generated during the industrialization of agricultural produce, as well as their high content of bioactive compounds with interesting functional properties, such as antioxidant and antibacterial properties, have encouraged the development of processes for their valorization, thereby contributing to the sustainability of this sector. However, the time-consuming and lab-intensive extraction protocols have severely hindered the application of these bioactive components in the food industry. As it is feasible to operate with environmentally friendly solvents, such as water, and, in some circumstances, without any solvent at all, using novel technologies for the extraction of bioactive components is a sustainable alternative. Moreover, these techniques are more rapid, with better extraction quality and efficacy. The food business is one of the industries that utilizes various extracts derived from these co-products, primarily in response to consumer demand for new goods with a lower synthetic preservative content generated through sustainable and eco-efficient techniques. Utilizing these co-products as a source of bioactive compounds and as an ingredient in numerous formulations for the food industry has become an attractive field today. Nonetheless, this procedure necessitates interdisciplinary research, which may include food chemistry, food technology, biotechnology, molecular biology, or toxicity.

## Figures and Tables

**Table 1:** Techniques for the extraction of bioactive components.

Technique	Advantages	Disadvantages	Bioactive Component	References
Maceration	Can be used for extracting thermolabile components. Cheap method	Lower extraction efficiency High extraction time Requires solvent in a larger volume	Polyphenols, anthocyanins, flavonoids, and essential oils	[59,60,61]
Percolation	More efficient than maceration	Lower extraction efficiency High extraction time	Alkaloids, Sterols, flavonoids, glycosides, saponins, phenols, lignins, sterols, and tannins	[62,63]
Decoction	More economical Only use water as a solvent Environment friendly	Effective only for heat-stable compounds Not suitable for light-sensitive compounds Heat and mass transfer efficiency is a crucial factor	Antioxidants and polyphenol	[64,65,66]
Reflux or solid–liquid extraction	Uses less solvent and has a shorter extraction time Easy High repeatability	Not suitable for volatile and heat-sensitive compounds	Essential oils, flavonoids, and polyphenols	[25,67,68]
Soxhlet extraction	High efficiency Low cost Basic technique and easy to use	Not suitable for volatile and heat-sensitive compounds Requires large quantities of solvents Sample preparation is time-consuming	Phenolics, antioxidants, essential oils, and flavonoids	[69,70,71,72]
Supercritical fluid extraction (SFE)	Greater penetration of the sample matrix and superior mass transfer compared with a liquid solvent Reduced extraction period Higher selectivity as the solvation power can be adjusted by altering the pressure and/or temperature. Ideal for extracting thermolabile compounds Minimal waste generation	Needs a sophisticated mechanism as a precise temperature and pressure should be maintained Not suitable for extracting polar compounds	Flavonoids, antioxidants, carotenoids, fatty acids, essential oils, terpenes, and polyphenols	[73]
Microwave-assisted extraction (MAE)	Reduced extraction time Lower solvent usage Cost-effective Better extraction yield compared with traditional methods	Not suitable for heat-sensitive compounds Not effective for non-polar compounds	Phenolic compounds, glycosides, flavonoids, terpenoids, essential oils, alkaloids, and saponins	[74,75]
Enzyme-assisted extraction (EAE)	Can be used to extract cell-wall-bound components Suitable for heat-sensitive materials Higher-quality extracts due to the high specificity and efficiency of enzymes Environmentally friendly	Not many enzymes have been studied and optimized for their extraction efficiency	Anthocyanins, polyphenols, carotene, terpenes, and flavonoids	[76,77]
Pulsed electric field extraction (PEFE)	Non-thermal technique. Minimal degradation of thermolabile compounds Can be used as a pre-treatment before conventional extraction Continuous method Short extraction time	Not suitable for products with high electrical conductivity as it reduces the resistance in the system	Phenols, flavonoids, proteins, anthocyanins, and carbohydrates	[78,79,80,81,82]
High-hydrostatic-pressure extraction	Low energy consumption High yield Effective in extracting both polar and non-polar compounds	Needs expensive equipment Need a lot of maintenance	Phenolic compounds, carotenoids, flavonoids, pectin, lutein, lycopene, and catechin	[83]
Ultrasound-assistedextraction (UAE)	Low energy consumption High yield Short processing time Can be used for heat-sensitive compounds	Can produce free radicals that will affect the quality of bioactive compounds Difficult to scale up for industrial uses	Phenolic compounds, flavonoids, oils, and anthocyanins	[58,84,85,86]

**Table 2:** Antioxidant activities of bioactive compounds extracted from various fruit wastes.

Sl No	Fruit Waste	Bioactive Compounds	Antioxidant Activity/Results	Reference
1	Mango waste	Catechin, epicatechin, andkaempferol.	A significant amount of these phenolic compounds contributes to the potential activity	[141]
2	Red pitaya seeds	Flavonoids and phenolic acids	The total phenolic content of the sample was found to be 13.56 ± 2.04 mg GAE/g dry weight	[142]
3	Pomegranate peels	Flavonoids and phenolic acids	Higher antioxidant activity in the peel than in the edible portions	[139]
4	Mango by-products	Phenolic acids, sterols, carotenoids, and tocopherols	A safer alternative to the synthetic antioxidants in biscuits, vegetable oils, and other different food formulations	[143]
5	Apple peel and seeds	Polyphenols and tannins	Superiority of bioactivity was observed in the case of peels compared with the seed portions	[130]
6	Citrus by-products	Flavonoids and phenolic acids	Depended on the species, cultivar, type of by-product, and harvesting conditions	[129]
7	Mango, papaya, and guava peels	Polyphenols	Antioxidant activities from the four assays indicated that mango peel extract possessed higher antioxidant properties.	[144]
8	Rambutan by-products	Phenolic acids and ellagitannins	Constituents contributed to the antioxidant potential of rinds	[145]
9	Pepper seed extracts	Capsaicin and di-hydrocapsaicin	Total polyphenolic content was 10.9 mg gallic acid equivalents/g residue	[146]
10	Plum, grapes, and elderberry fruit by-products	Anthocyanins	The highest values of 90.19 and 89.86% were attributed to elderberry fruit and Italian red grape extracts respectively	[147]
11	Pomegranate by-products	Flavonoids and condensed and hydrolyzabletannins	Bioactive compounds found in by-products have antioxidant properties that help protect cells from various stimuli-induced oxidative stresses and cell death	[148]
12	Orange by-products	Ascorbic acid, flavonoids, and phenylpropanoids	Flavonoids are an important subgroup exhibiting high antioxidant activity	[149]
13	Grape seeds	Phenolic acids and flavonoids	Higher polyphenol concentration and antioxidant potential of the sample when compared with bagasse extract	[103]

**Table 3:** Antimicrobial properties of bioactive compounds extracted from various fruit wastes.

Sl No	Fruit Waste	Observation	Reference
1	Citrus essential oil	Antimicrobial activity against species such as *Trichoderma viride*, *Cladosporium herbarum,* and *Aspergillus flavus*	[138]
2	Plum, grapes, and elderberry fruit by-products	Constituted sizeable contents of anthocyanins and significantly inhibited the growth of *B. cereus*	[147]
3	Grape by-products	Antimicrobial activities of winemaking by-products were verified against foodborne pathogens, with the lowest MICs for Gram-positive bacteria and medium influences on the MICs of Gram-negative bacteria	[159]
4	Muscadine grapes	Muscadine polyphenols at 4 × minimum inhibitory concentration caused nearly a 5 log10 CFU/mL decrease in cell viability for *S. aureus* in 6 h with lysis	[152]
5	Banana peels	The antimicrobial potential was due to the presence of tannins and phenolics	[157]
6	Mango kernel extracts	Greater inhibition against *S. aureas* at various concentrations than against *E. coli*	[154]
7	Orange and pineapple peels	The pineapple sample showed the largest zone of inhibition against Klebsiella and the smallest against *Bacillus subtilis*	[151]
8	Pomegranate by-products	Peel extract displayed excellent antioxidant activity, while the seed extract did not have any substantial activity	[158]
9	Mandarin, broccoli, and orange by-products	All samples showed inhibitory effects against *Salmonella* spp., *Escherichia coli*, *Bacillus cereus*, and *Listeria monocytogenes.*	[150]
10	Orange, banana, and lemon peels	Effectiveness was found to be higher in yellow lemon, followed by orange and banana peels. *Klebsiella spp.* showed the highest sensitivity to the extract of yellow lemon peel and showed the largest zone of inhibition	[160]
11	Quince fruit peel	Effective against bacteria growth owing to flavonoid proportions in the peel in conjunction with chlorogenic acid	[161]

**Table 4:** Bioactive compounds extracted from fruit waste and their application as a natural food preservative.

Food Waste/Bioactive Compound	Food Preservation Effect	Reference
Apple pomace	Inhibitory effect against pathogens *Helicobacter pylori*	[221]
Kiwi leaves (alcoholic and hydroalcoholic extracts)	Antimicrobial effect against *S. aureus*	[222]
Olive mill wastewater (phenols)	Antimicrobial action against *E. coli*, *P. aeruginosa*, *S. aureus*, and *B. subtilis* strains	[223]
Tomato wastes	Antimicrobial activity of tomato waste extracts against *S. aureus* correlated moderately with isochlorogenic acid content	[224]
Acetone and methanol carrot peel extracts	Growth inhibition of *Shigella flexneri, E.coli, S. aureus,* and *Klebsiella pneumoniae*	[225]
Jabuticaba seeds	Ellagitannins and ellagic acid in the extracts contained antimicrobial and antioxidant properties.	[226]

## Data Availability

The data presented in this study are available on request from the corresponding author.
